# Neuronal Correlates of Empathy: A Systematic Review of Event-Related Potentials Studies in Perceptual Tasks

**DOI:** 10.3390/brainsci14050504

**Published:** 2024-05-16

**Authors:** Rita Almeida, Catarina Prata, Mariana R. Pereira, Fernando Barbosa, Fernando Ferreira-Santos

**Affiliations:** 1 Laboratory of Neuropsychophysiology, Faculty of Psychology and Education Sciences, University of Porto, 4200-135 Porto, Portugal; almeida.c.rita@gmail.com (R.A.);; 2 GOS Institute of Child Health (UCL, UK), London WC1N 1EH, UK

**Keywords:** empathy, perception, EEG/ERP, affective pictures, facial stimuli, mental states, social language

## Abstract

Empathy is a crucial component to infer and understand others’ emotions. However, a synthesis of studies regarding empathy and its neuronal correlates in perceptual tasks using event-related potentials (ERPs) has yet to occur. The current systematic review aimed to provide that overview. Upon bibliographic research, 30 studies featuring empathy assessments and at least one perceptual task measuring ERP components in healthy participants were included. Four main focus categories were identified, as follows: Affective Pictures, Facial Stimuli, Mental States, and Social Language. The Late Positive Potential was the most analyzed in Affective Pictures and was reported to be positively correlated with cognitive and affective empathy, along with other late components. In contrast, for Facial Stimuli, early components presented significant correlations with empathy scales. Particularly, the N170 presented negative correlations with cognitive and affective empathy. Finally, augmented N400 was suggested to be associated with higher empathy scores in the Mental States and Social Language categories. These findings highlight the relevance of early perceptual stages of empathic processing and how different EEG/ERP methodologies provide relevant information.

## 1. Introduction

Empathy is significantly relevant in daily life, even if individuals are not aware. When seeing an accident on the news, hearing a friend share a story, or reading a book, people infer what the other person is feeling or thinking. Evidence shows that empathic abilities start developing in early childhood [1,2,3] and continue to do so through adolescence [4]. Despite this pertinent presence in life, there is a longstanding debate regarding the concept of empathy and the methodologies that can be used to measure it [5].

Hall and Schwartz [6] conducted a review regarding the concept of empathy in 489 studies. They highlighted that those studies considered different definitions of empathy—some focused on empathy as a trait oriented to people in general, while others explored specific contexts, such as empathy scores in victims of sexual abuse. Some used self-report measures and others relied on informant reports. Seventy-two different instruments were used in total and the most used instrument, the Interpersonal Reactivity Index (IRI) [7], was only included in 36% of the studies. The discrepancies continue to be described on several levels and provide evidence that, as the authors accurately describe it, “the continued vague use of the term empathy to characterize a wide range of different methods and definitions can only dilute the value of scholarship” (p. 28).

Considering these different perspectives, Decety and Jackson [8] suggested that the multidimensional nature of empathy requires an equally multidimensional conceptualization. Thus, empathy can be defined as a multidimensional construct, involving a variety of psychological processes, with the following three fundamental aspects: (i) the ability to share another person’s emotional state; (ii) mentalizing, which refers to the ability to understand another person’s psychological state and their cognitive processes; and (iii) prosocial concern, which consists of expressing motivation to help others [8,9]. This multidimensional approach provides a common ground for the different definitions, some of which favor the affective aspects, others the cognitive, and several focus on both [10]. Despite these different perspectives, a review conducted by Eklund and Meranius [11] supports the idea that it is possible to find four common aspects across empathy conceptualizations, as follows: “the empathizer (1) understands, (2) feels, and (3) shares another person’s world (4) with self-other differentiation” (p. 6). These similarities constitute a step towards a more inclusive and comprehensive conceptualization.

Traditionally, research on empathy emerged primarily from developmental and social psychology [8], but there has been an increase in neuroscience-based approaches to this construct in the last two decades, due to the improved technology, methods, and understanding of neuronal function [11]. The application of techniques such as functional Magnetic Resonance Imaging (fMRI) and Event-Related Potentials (ERPs)—electrophysiological measures of cortical activity that reflect changes in response to a discrete stimulus [12]—have enabled a better understanding of the brain regions and neuronal mechanisms underlying empathy. For instance, Kogler et al. [13] utilized fMRI data to support the existence of two distinct pathways associated with empathy—one linked to affective empathy and another to cognitive empathy. Additionally, neuroscience research suggested that a complete empathic experience only occurs when both neuronal pathways are simultaneously active [14,15]. These findings are aligned with the multidimensional conceptualization of empathy described above and highlight the complexity of the processes involved in this construct.

Although ERP studies provide limited information on the spatial locus of neuronal activity, they allow the recording of brain activity with a millisecond temporal resolution [16], providing information on fast-occurring neuronal processes that are not captured using fMRI. Given the key role that empathy has in social, personal [17,18], and professional contexts [19,20], ERP studies can be a highly relevant source of evidence on the neuronal bases of empathy. Through ERP techniques, researchers can gain insights and better understand the neural networks that underlie the construct of empathy, as well as how they are modulated by various factors. 

However, one of the major problems in this field of research is the large variability in tasks and research design, which renders comparisons between studies difficult to perform and interpret. For example, in the four ERP studies on empathy cited in the previous paragraph, five different ERP waves or components are reported to be associated with empathy—Early Posterior Negativity (EPN), N110, N200, P300, and Late Positive Potential (LPP). High variability is also present, even when studies focus on a single ERP component. Indeed, a meta-analysis including 125 studies regarding empathy and ERN (Error-related Negativity) conducted by Amiruddin et al. [21] indicated no overall significant association between empathy and ERN, but also a significant heterogeneity across studies. Adding to the issues of conceptual and methodological heterogeneity between studies, there is also diversity regarding the EEG equipment and recording settings, as well as signal processing and data analysis procedures. 

As such, it is important to collect data regarding the wide range of ERP components reported in empathy research and consider what each potential can indicate concerning the processes that are occurring, as well as the time frame in which they occur. For example, the N170 occurs as an early negative deflection around 170 ms, which is larger for face stimuli [22], while the LPP is a slow sustained positivity wave that occurs at 400–600 ms in centro-parietal sites, suggested to reflect sustained attention and motivational significance [23]. This information, associated with the task applied, allows for a better understanding of what each ERP component maps, in terms of the concepts of empathy discussed above.

In the present study, to provide clarity, we will consider early and late ERP components separately. Early components are usually associated with stimuli characteristics and low-level basic sensory processing and perception. They present an automatic nature, but may be modulated by top-down processes. Late components tend to reflect higher and conscious levels of cognitive processing [16,24,25]. Depending on the context of interest, studies may have a greater focus on lower-level stimuli characteristics and sensory processing, such as research regarding facial perception or emotional visualization, or be oriented to more high-level cognitive processes, such as decision-making [12]. 

Decety and Lamm [26] suggest a model explaining empathy as a feedback loop, where bottom-up processes—such as the perception of others—feed information coming from external cues to top-down processes. Top-down processes, in turn, provide regulation and control over those low-level inputs, providing flexibility and less dependence on external cues. This indicates that bottom-up processes are responsible for direct emotion sharing that is automatically initiated by perceptual inputs. In accordance with this, other perception–action empathy models support the idea that observing another person expressing a certain emotion activates a shared representation and autonomic response in the individual, which can then be acted upon or inhibited [27]. Furthermore, in line with this model and previous examples, neuroimaging studies have shown that, even if certain brain regions are consistently activated during empathic tasks, there are distinct areas of the brain that are only active when engaging in what they specifically call affective–perceptual forms of empathy versus cognitive–evaluative forms of empathy [28].

This highlights the importance of the perceptual states of empathic processing, which, according to the literature, are automatically present and provide the core information for the rest of the top-down modulation and the settlement of priors [26,27]. Taking this into account, exploring studies with perceptual approaches appears to be an essential starting point for understanding the empathic processes and how different ERP components are involved. Studies focused on tasks such as decision-making or gambling involve more cognitive and complex processes [15,29], which are dependent on the earlier perceptual empathic processes we are looking to explore. Likewise, research shows that processes such as empathy for pain involve the activation of the cerebral structures as the experience of self-pain [30] and depend on several points of elicitation, such as concrete or abstract stimuli and the social situation in which the participants are included, involving higher processes [31]. Furthermore, this overview on empathy for pain has already been reviewed and meta-analyzed [32,33] and, as such, will not be included in this review. The present study will include only tasks that emphasize perceptual processing, involved in recognizing, interpreting, and making sense of sensory information from the environment [34,35]. These tasks rely primarily on sensory stimuli and their characteristics—such as faces, depictive scenes, tactile sensations, auditory tones, and contextual semantics [12]. As such, perceptual tasks address not only the visualization of affective stimuli, but also the participants’ perception of other external sensory cues (e.g., linguistic aspects or observed actions).

In sum, several sources of heterogeneity (e.g., conceptual, methodological, and technical) pose significant barriers to synthesizing the ERP findings regarding empathy. To address this issue, we conducted a systematic review of relevant research employing ERP measures to study the perceptual aspects of empathy. In line with this, the review has two aims: (1) to explore the designs (including task structure, stimuli, and measures) and ERP components that have been studied, as well as (2) to explore the results and conclusions achieved in the studies included. The present review provides a compilation and integration of these aspects, allowing the identification of the main similarities and gaps.

## 2. Materials and Methods

### 2.1. Search Strategy and Study Selection

The present review was conducted considering the PRISMA Statement [36]. A systematic search was first conducted in March 2020, on scientific databases available through EBSCOhost, PubMed, and Web of Knowledge, using the following search expression: (“empathy” OR “empath*”) AND (“erp” OR “event related potential*” OR “evoked potential*”). A broader search expression was used to incorporate a wider range of papers exploring both low- and high-order tasks, such as perceptive and decision-making tasks, respectively, even though the present review was only focused on tasks regarding perceptual aspects of external stimuli.

**Figure 1:**
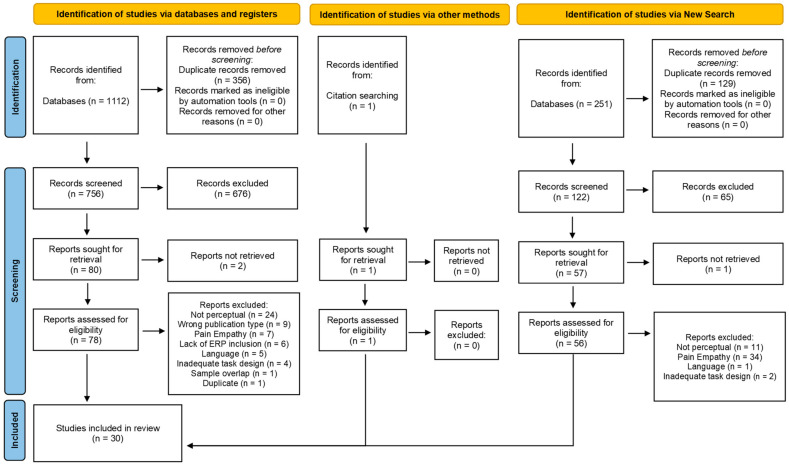
PRISMA 2020 flow diagram for new systematic reviews, which included searches of databases, registers, and other sources.

The search was limited to titles, abstracts, and keywords published in English. After duplicated entries were removed, 755 studies were considered potentially relevant. One study was further identified through citation searching, resulting in a total of 756 studies. The PRISMA flow diagram of this review is presented in Figure 1. The search was replicated in April 2023 and, once duplicates were removed, 122 new articles were included for screening.

The Quantitative Research Assessment Tool [37] was used to assess the quality of the final studies. All studies presented scores greater than zero and 50% of the studies scored six or higher, so study quality was ensured. This study was not registered on Prospero.

### 2.2. Inclusion and Exclusion Criteria

Studies were considered eligible if they were in accordance with the following inclusion criteria: (a) study reported data on healthy participants/controls; (b) study contained at least one perceptual task to assess ERP amplitude and/or latency; and (c) study contained at least one empathy measure, either through the EEG task or scores of self-reported instruments analyzed with EEG data.

Studies were excluded if (a) the study was a commentary, a case report, and/or did not report any quantitative findings; and (b) the studies included the same participants and reported overlapping results as other studies already included in the review (in these cases, we excluded all articles except the most complete).

### 2.3. Primary Screening

Two independent reviewers (RA and CP) conducted a primary screening of titles and abstracts. Inter-rater agreement between reviewers was substantial (Cohen’s κ = 0.79). Discrepancies were resolved by a third reviewer (MRP). This led to the exclusion of 741 out-of-topic studies and 135 studies retained for eligibility assessment through full-text reading (Figure 1).

### 2.4. Data Extraction

Data extraction was conducted for the following variables:

**Sample and Measures.** Size and group information were coded, including age, sex, handedness, and pathology. Whenever possible, the final sample size (after exclusions and EEG data treatment) was included. Empathy measures/instruments and respective means and standard deviations were included when reported.

**Stimuli and Tasks.** Stimuli coding was conducted regarding type (e.g., image, video), content description, kind (e.g., natural, cartoon), dataset, color (black and white/color), and image size. Tasks were coded according to the adequate nomenclature and regarding type (active/passive), emotion (explicit/implicit), number of conditions, stimuli duration, number of stimuli per condition, and number of trials per condition.

**EEG Recording and Pre-Processing.** EEG channel montage, number of channels, online recording reference, and offline reference were included. Information regarding applied filters, as well as methods for artifact rejections were also extracted.

**Event-Related Potentials**. ERP components reported in the study were coded for name, time window of measurement, electrodes, hemisphere, scoring method, amplitude, and latency—when available. Plot inclusion (yes/no) and type of plot (e.g., scatterplot, table, grand-average waveform) were also included.

**Statistical Analysis and Results.** Statistical analysis conducted and description, effects, and statistics (e.g., *p*-value, r) were included for amplitude (mean, peak, or peak-to-peak amplitudes) and latency for each condition and component included.

Two reviewers performed data extraction independently, one for all selected studies retained for eligibility assessment through full-text reading (RA) and the other (CP) for a sample of 11% (*n* = 15) of the studies. Coding agreement across all selected variables was 76%. Discrepancies were resolved by consensus, which resulted in complete agreement. A total of 105 articles were then excluded according to the inclusion and exclusion criteria (due to information uncovered during data extraction), leading to the final inclusion of 30 papers of interest.

## 3. Results

Data were extracted from 30 studies published between 2011 and 2023. A summary of the included studies can be seen in Table 1.

### 3.1. Sample

A total of 1140 participants were included (618 women, 54%) and participants’ age ranged from 4 to 75 years, with a minimum mean age of 18.50 (*SD* = 3.74) and a maximum mean age of 34.10 years (*SD* = 14.60). Ten studies (33%) did not report age range.

Sample size varied between 15 and 102 participants (*M* = 38.00, *SD* = 19.16). Only one study included children [38], from ages 4 to 6, while the rest of the studies included only adults. One study included low- and high-empathy groups [39], while Tobón et al. [40] added a third control group with “normal”-empathy and two groups of ex-combatants with normal and poor empathy. In this case, normal-empathy groups were organized considering a cluster analysis of Interpersonal Reactivity Index scores (IRI) [7] and presented average to high scores. Three studies considered the differences between men and women [41,42,43], while another two studies [44,45] had a typical control group and a group of “neglectful mothers”, drawn from a pool of at-risk mothers. Both studies included the same “mothers” sample, but applied different tasks, and had a two-week interval between studies. Another study included young adults, middle-aged adults, and older adults [46]. There was also the inclusion of frequent and infrequent players of violent games [47], as well as an ADHD group and a typical control group [48]. Altavilla et al. [49] divided their sample into a control and introspection group, in which the latter received an introspective writing task for 7 days, while the control group only had to describe their days.

**Table 1:** Summary of the main characteristics and results of the studies included in the review.

Task Category	Study	Group	Sample Size	Empathy Measure	Task	Stimulus Type	ERP	Statistical Analysis	Results
Affective Pictures	Althaus et al. [50]	Adults	52	IRI EQ	Affective Visualization	Emotional Images	LPP	Correlation; Stepwise Multiple Regression	**LPP:** Positive correlation with IRI-PD for “Human Effect” (P3, P7); amplitude predicted by IRI-PD for “Human Effect” (P3).
	Balconi et al. [51]	Adults	15	BEES IRI	Affective Visualization	Emotional interaction scenes	N300 P300	Correlation	**N300:** Positive correlation with BEES for negative condition.
	Groen et al. [41]	Men Women	27 15	IRI EQ	Emotional Visualization	Emotional scenes	N100 N200 LPP	Correlation	**N2:** Positive correlation with IRI-EC in “Human Effect” (Cp3). **LPP:** Positive correlation with IRI-EC in “Human Effect” (P3).
	Leon et al. [44]	Control Mothers Neglectful Mothers	14 14	IRI	Emotional Rating	Emotional images	EPN P200 LPP	ANCOVA Correlation	**No Effects**
	Luo et al. [52]	Adults	34	None	Affective Visualization	Images of different scenes	N200 LPP	ANOVA	**N2:** At frontal and central sites, highly negative (HN) stimuli elicited a more positive shift than moderately negative (MN) stimuli. At parietal sites, HN stimuli elicited smaller negative deflection than MN and neutral stimuli. **LPP:** At central sites, HN stimuli elicited a larger amplitude than MN and neutral stimuli, in both men and women. At parietal sites, for women, the HN amplitude was larger than that of MN and neutral stimuli. For men, HN stimuli elicited larger LPP amplitudes than MN and neutral stimuli.
	Kanske et al. [53]	Adults	27	IRI	Attentional Blink	Emotional images	P300	Correlation	**P3:** Positive correlation with IRI-Total, IRI-PT, and IRI-F for negative-neutral and positive-neutral conditions.
	Romeo and Spironelli [54]	Adults	40	IRI	Passive Viewing	Emotional Images	P100 P300	ANCOVA	**No Effects**
	Tobón et al. [40]	Ex-combatants—normal empathy Ex-combatants—poor empathy Non-ex-combatants—normal empathy	20 20 20	IRI	Scene Classification	Pictures of social situations	EPN LPP	ANOVA; Correlation	**LPP:** Non-ex-combatants presented smaller amplitudes than the other groups; negative correlation with IRI-PD.
Facial Stimuli	Altavilla et al. [49]	Control Introspection	14 15	None	Reading the Mind in the Eyes	Eye Expressions and Objects	P200 P300 LC1 LC2	ANOVA	**P200:** No Effect. **P300:** Larger P300 to eye expressions in introspection group after 7-day writing task. **LC1:** No Effect. **LC2:** No Effect.
	Balconi and Canavesio [39]	Low Empathy High Empathy	14 14	BEES	Affective Visualization	Facial expressions of emotion	N200	ANOVA	**N200:** The high-empathy group presented increased amplitude and larger amplitude than the low-empathy group in response to anger, fear, and happiness.
	Bauser et al. [55]	Adults	17	BEES IRI	Faces Affect Identification	Frontal and averted Bodies Frontal and averted Faces	P100 N170	Correlation	**P1 latency:** Negative correlation with IRI-PT for angry averted bodies and happy averted faces; with IRI-EC and IRI-PD for neutral frontal faces. **N170:** Positive correlation with IRI-PD for frontal angry bodies and averted happy bodies; negative correlation with IRI-EC for angry frontal/averted faces and neutral averted faces; negative correlation between latency and IRI-F for neutral averted faces.
	Choi and Watanuki [56]	Adults	32	IRI	Oddball	Facial expressions of emotion Flowers	Early LPP Late LPP	Correlation	**Early LPP:** Positive correlation with IRI-Total for face targets (Pz). **Late LPP:** Positive correlation with IRI-F for face targets (Cz, Pz) and non-targets (Pz); positive correlation with IRI-Total for face targets (Pz).
	Choi et al. [57]	Adults	22	IRI	Oddball	Facial expressions of emotion	N170 Early LPP Late LPP	Correlation	**N170 (T6):** Negative correlation with IRI-PT for happy and surprised faces; with IRI-EC for happy, angry, surprised, and afraid faces; with IRI-Total for angry and surprised faces. **Early LPP (Pz):** Positive correlation with IRI-PT for angry and afraid faces. **Late LPP (Fz):** Positive correlation with IRI-Total for happy, angry, surprised, and sad faces; with IRI-PT for happy, surprised, and afraid faces; with IRI-F for angry faces.
	Clark et al. [18]	Adults	42	EQ	Face Processing Task	Facial Expressions and Characterization Stories	N170 EPN LPP	Linear Mixed Model	**N170:** Tendency to become larger (more negative) with increasing empathy. **EPN:** Larger for subjects with very low or very high EQ scores; larger for unintentionally negative characters in lower EQ scores and for neutral characters in higher EQ scores. **LPP:** No effects
	Dozolme et al. [58]	Adults	32	EQ	Facial Congruence	Synthetic facial expressions of emotion and sentences	P100 N170 N400 LPP	Regression	**N400:** Higher cognitive empathy (EQ) predicted more negative amplitudes for incongruent stimuli (left occipital region); larger amplitude for congruent faces—corresponding to more negative components for the incongruent conditions. **LPP:** Larger amplitude at the frontal midline and dorso-frontal regions for incongruent faces.
	Fernandes et al. [46]	Young Adults Middle-aged Adults Older Adults	30 30 29	None	Facial Congruence	Congruent or incongruent FEE	N170 Early LPP Late LPP	ANOVA; Correlation	**N170:** Larger for incongruent than congruent conditions for fear, in older adults. **LPP:** Higher (early and late) amplitude for congruent than incongruent in younger and middle-aged adults.
	Lazar et al. [59]	Adults	57	EQ	ERP faces–houses	Houses and faces with neutral expressions	N170	Stepwise multiple regression	**N170:** Higher EQ predicts decreased amplitude (P8).
	Luo et al. [42]	Men Women	16 16	IRI	Emotional categorization (Other-Task) Emotional categorization (Self-Task)	Facial expressions of emotion	P200 LPC	Correlation	**P200:** Positive correlation with IRI-PT in self-task (C4). **LPC:** Negative correlation with IRI-F (C4) and IRI-PT (CPz—other-task; Fz, FCz—self-task); Positive correlation with IRI-PT score in other-task (C4).
	McCrakin and Itier [60]	Adults	44	TEQ	Eye-Tracking Facial Task	Eye Gaze associated with Positive, Negative, and Neutral Sentences	N100 N170 N200 EPN	ANOVA	**N100:** Larger for averted gaze in negative empathy and larger for direct gaze in positive empathy. **N170:** No Effect. **N200:** Larger for higher empathy scores and for neutral than positive and negative empathy conditions **EPN:** Larger for negative than neutral empathy condition (right hemisphere).
	Naumann et al. [38]	Children	31	EMK 3–6	Delayed Match-to-Sample Task	Facial Expressions of Emotion	P100 N170 P300	Correlation	**No effects**
	Rodrigo et al. [45]	Control Mothers Neglectful Mothers	14 14	IRI	Emotional Categorization	Infant Facial Expressions	N170 P200 LPP	Regression	**No significant effects**
	Stockdale et al. [47]	Frequent players of violent video games Infrequent players of violent video games	30 31	IRI	Stop-signal	Facial Expressions of Emotion	P100 N170 N2/P3 Complex	Moderation	**P1:** Reduced amplitude for happy faces in frequent players at lower empathy levels; empathy negatively predicts P100 amplitude for happy faces in infrequent players.
	Thoma et al. [48]	ADHD Control	18 25	IRI EQ	Faces Affect Identification Task	Upright and Inverted Bodies and Faces	N170	Correlation	**No effect.**
Mental States	Albrecht and Bellebaum [61]	Adults	33	EQ	False-belief Task	Two “shells” with a hidden ball	Early negative FC component Late negative FC component	Linear Mixed Effects	**Early negative FC:** In high empathy participants, larger for correct than error in trick condition and the opposite for no-trick condition (only in low difficulty). **Late Negative FC:** No effects.
	Albrecht and Bellebaum [62]	Adults	102	EQ	False-belief Task	Two “shells” with a hidden ball	Late negative FC component	Linear Mixed Effects	**Late Negative FC:** Larger for correct than error in trick condition and the opposite for no-trick condition.
	Ferguson et al. [63]	Adults	28	EQ	Belief consistency	Action description —hiding a target object	N400	Correlation	**N400:** Negative correlation with EQ for inconsistency effect.
	Manfredi et al. [64]	Adults	31	EI	Humorous ToM	Comic grey-scale panels from Monica’s Gang™ comics	N400 LP	ANOVA; Correlation	**N400:** Positive correlation with empathy scores in incongruent strips; larger amplitude for incongruent, humorous non-ToM, and humorous ToM than congruent strips **LP:** Positive correlation with empathy scores in incongruent and ToM strips; larger amplitude to humorous ToM strips than congruent and incongruent strips.
Social Language	Jiang and Pell [65]	Adults	30	IRI	Vocal Utterance	Audio sentences	N100 P200 N400 Delayed, sustained positivity	Linear Mixed Effects	**N400:** Reduced amplitude for vocal expressions after lexical phrases in subjects with higher empathy scores **Delayed, sustained positivity (900–1250 ms and 1250–1600 ms):** Participants with higher IRI-Total presented a larger response to lack of confidence.
	Jiang and Zhou [66]	Adults	32	EQ	Rapid Serial Visual Presentation	Conversational scenarios (Sentences)	N400 Late Positivity Delayed, Sustained Positivity Late Anterior Negativity	ANCOVA; Linear Regression	**N400:** EQ predicted the N400 difference between Referent and Ambiguous conditions and between Referent and Status conditions; participants with higher empathy showed larger N400 effects in Referent condition. **LP:** Larger amplitude in subjects with higher EQ scores in Status condition. **Delayed Sustained Positivity:** Larger amplitude in subjects with higher EQ scores in Status condition. **Late Anterior Negativity:** EQ score predicted the difference between the Status and Referent condition.
	van den Brink et al. [43]	Men Women	12 15	EQ	Social Language	Audio sentences with inferred information	N400	Correlation; Multiple Regression	**N400:** Higher EQ scores revealed larger speaker identity N400 effects; EQ predicts the speaker identity N400 effect.

Note: Empathy Measure—IRI = Interpersonal Reactivity Index; EQ = Empathy Quotient; BEES = Balanced Emotional Empathy Scale; TEQ = Toronto Empathy Questionnaire; EMK 3–6 = Inventory to survey of emotional competences for three-to-six-year-olds; EI = Brazilian Empathy Inventory; ERP Components—LPP = Late Positive Potential; EPN = Early Posterior Negativity; LC = Late Component; LPC = Late Positive Complex; FC = Frontocentral; LP = Late Positivity; Results—PT—Perspective Taking; F—Fantasy; EC—Empathic Concern; PD—Personal Distress.

### 3.2. Instruments

Six different empathy instruments were used in the included studies. The Interpersonal Reactivity Index (IRI) [7] was the most widely applied instrument, included in a total of 15 studies (50%). This instrument presents four distinct subscales—Perspective-Taking (PT), Fantasy (FS), Empathic Concern (EC), and Personal Distress (PD). It was the single empathy scale in 10 studies and was combined with others in five studies. The second most common instrument was the Empathy Quotient (EQ) [67] (*k* = 11), while the Balanced Emotional Empathy Scale (BEES) [68] was only included in three studies. Other instruments reported were only used in one study each—the Brazilian Empathy Inventory (EI) [69]; the Inventory to survey emotional competencies for three-to-six-year-olds [70]; and the Toronto Empathy Questionnaire (TEQ) [71].

Most studies considered only one empathy instrument (*k* = 22). Five studies included two instruments. The IRI plus EQ combination was the most included (*k* = 3). Three studies (10%) did not include any instrument to measure empathy, assessing it through experimental tasks.

### 3.3. Task Design

Regarding task design, four main categories were identified by consensus (RA and FFS): (1) Affective Pictures (*k* = 8); (2) Facial Stimuli (*k* = 15); (3) Mental States (*k* = 4); and (4) Social Language (*k* = 3). Only three studies applied two tasks, all associated with facial expressions of emotion [48,52,56]. The remaining studies (90%) included one task. Regarding task type, 26 studies included active tasks and four included passive tasks.

### 3.4. Stimuli

Two studies used audio stimuli with inferences regarding the speaker’s characteristics [43,65], while the other used visual stimuli. The stimuli consisted mainly of facial expressions of emotion (*k* = 12) or eye gaze (*k* = 2), emotion-inducing images (*k* = 8), and comic grey-scale panels (*k* = 1). Three studies used sentences or images describing true or false beliefs of an observed person [61,62,63].

Regarding stimuli kind, most were natural (83%) and the others varied among schematic or avatar faces and cartoon panels. Eighteen studies included stimuli of color, eight included black and white or grey stimuli, and one used both black and white and color stimuli.

Only 19 studies (63%) reported the dataset from which the stimuli were extracted, in a total of nine different datasets. Eight studies used the International Affective Picture System (IAPS) [72]. The remaining studies reported different datasets, such as the Karolinska Directed Emotional Faces [73] (*k* = 2); the NimStim set of facial expressions [74] (*k* = 2); the Radboud database [75] (*k* = 2); and the Bochum Emotional Stimulus Set (BESST) [76] (*k* = 2).

### 3.5. EEG Recording and Preprocessing

EEG data were recorded from 64 electrodes in 30% of the studies (*k* = 9) and only two studies utilized more than 64 electrodes (128 in both cases). One study did not report this information.

Regarding EEG online reference, the mastoids (either left, right, or both) were the most selected (*k* = 11), followed by studies using the earlobes (*k* = 6), the Cz electrode (*k* = 6), the FCz electrode (*k* = 4), or the nose (*k* = 1). Two did not report this information. As for offline reference, the most common procedure was to compute the electrode average (*k* = 16), followed by the mastoids (*k* = 8), earlobes (*k* = 4), and Cz (*k* = 1). One study did not report this information.

Regarding preprocessing information, only one study did not report any high-pass filter. Overall, the applied filters ranged from 0.01 to 1 Hz and the most referred were 0.01 Hz (*k* = 9) and 0.05 Hz (*k* = 5). As for low-pass filters, they varied from 20 to 125 Hz and the most common was 30 Hz (*k* = 12), followed by 100 Hz (*k* = 6). Twenty-two studies did not use any other filter, while the ones reported ranged from 60 to 200 Hz. Only three studies reported using a notch filter (50 Hz).

Regarding artifact rejection, 15 studies (50%) reported that EEG data were submitted to an independent component analysis (ICA) [77], while the rest of the studies do not mention it. Furthermore, rejection based on visual inspection was also reported (*k* = 13), as well as automatic threshold rejection (*k* = 22) with values varying between 50 and 200 μV. Only eight studies did not report the number or percentage of final trials removed.

### 3.6. Event-Related Potential Components

The number of ERP components varied across studies, ranging from one to five per study. Eight studies (27%) reported only one ERP component and nine reported two, followed by eight studies (27%) that reported three. In total, 72 ERP measures were analyzed, corresponding to 23 distinct components. As shown in Table 1, the most widely analyzed potentials were the LPP (*k* = 12), N170 (*k* = 11), N400 (*k* = 6); P300 (*k* = 6), P200 (*k* = 5), and P100 (*k* = 5).

Components were quantified using mean amplitude in the selected time window in most studies (*k* = 19), while the rest reported peak amplitude (*k* = 6), peak-to-peak (*k* = 2), or baseline-to-peak (*k* = 1) amplitude. Two studies considered both peak and mean amplitude in different ERP components [18,46].

Twenty-three studies included plots with ERP and empathy association. The most included plots were grand-average waveforms of the components (*k* = 12), scatterplots (*k* = 9), and description tables (*k* = 8).

### 3.7. Findings

Due to the number of ERP components and studies included in this review, results will be presented by task design category, providing a more cohesive report. Information regarding all results can be found in Table 1.

#### 3.7.1. Affective Pictures

Eight studies focused on the visualization of emotion-inducing images with distinct valence. Despite slight differences regarding task details, all intended to explore how empathy is related to the perception of stimuli valence—positive, negative, and neutral. In two studies, the goal was to assess how valence perception can be distinct between images with only humans, images without humans, or those with human–animal interaction, and how this distinction is correlated to empathy. The IAPS [72] was used in six of the studies, providing a common dataset for most studies in this category. Information regarding the results for this category can be found in Table 2.

##### Early Components–N100, P100, N200, P200, and EPN

For the N100, Groen et al. [41] used positive, negative, and neutral images depicting humans versus without humans—we will refer to this contrast as the “human effect”. No significant correlation between N100 amplitude and EQ [67] or IRI [7] scales was found for any stimuli.

The P100 component was analyzed by Romeo and Spironelli [54] through the visualization of images of positive categories (sports and erotic), negative categories (mutilation and fear), and neutral. Using the IRI-PD scores as a covariate, the authors found no significant effect regarding this component.

Moreover, Groen et al. [41] found that N200 amplitude was positively correlated with the Empathic Concern subscale of the IRI for the “human effect” in positive emotions, as well as for the “human effect” in negative emotions. The N200 is a negative component and was presented with negative values, so this positive correlation indicates that higher empathy scores are associated with smaller amplitudes. Luo et al. [52] explored empathy through a task with pictures of humans in negative and neutral contexts to uncover differences in affective empathic responses. The N200 was reported to be smaller for highly negative stimuli than for moderately negative and neutral stimuli, respectively, at frontal and central sites, as well as for neutral stimuli at parietal sites, in both women and men—indicating an effective response to others’ distress.

Regarding the P200 component, no significant correlations were found for the IRI subscales and emotional images [44].

For the EPN, Leon et al. [44] did not find, once again, any significant correlations with the IRI subscales. This is mostly in accordance with the reports from Tobón et al. [40], who found no correlation between EPN amplitude and IRI scores and no difference between empathy groups.

##### Late Components–N300, P300, and LPP

The N300 amplitude was found to be positively correlated with BEES scores for negative stimuli both for human and human–animal interaction. No significant correlation was found for the IRI subscales [51].

Regarding P300 amplitude, Balconi et al. [51] reported no significant correlation with IRI and BEES scores, while Kanske et al. [53] reported a positive correlation with IRI-Total, IRI-PT, and IRI-F for negative minus neutral stimuli and positive minus neutral stimuli difference waves. However, Romeo and Spironelli [54] (2023) found no significant effect while using the IRI-PD score as a covariate in the visualization of positive, negative, and neutral images.

The LPP was the most explored component in the Affective Pictures category (*k* = 5). Several significant correlations were found with the IRI subscales. The IRI-PD subscale was reported to be positively correlated with LPP amplitude for “human effect” [41,50] and “human effect” in negative emotions [41]. However, a negative correlation with the IRI-PD was referred to by Tobón et al. [40], who also reported that a control group compared with two ex-combatants groups, with higher empathy scores, presented smaller amplitudes. The IRI-EC was also reported to be positively correlated with a “human effect” on positive emotions only [41]. Leon et al. [44] reported no significant correlation between LPP amplitude and IRI scores, while Luo et al. [52] reported larger amplitudes for highly negative stimuli than for moderately negative and neutral stimuli.

#### 3.7.2. Facial Stimuli

Fifteen studies related to Facial Stimuli focused mainly on the distinction of facial expressions of emotion (FEE), the processing of faces versus bodies/houses/flowers, eye gaze, and gender bias. Information regarding the results for this category can be found in Table 3.

##### Early Components–N100, P100, N170, N200, P200, and EPN

Only one study [60] in this category explored the N100. The task consisted of the presentation of sentences previously rated as positive (e.g., using the verb “saved”), neutral (e.g., “fed”), and negative empathy (e.g., “killed), followed by face pictures that presented either a directed or averted eye gaze. The participants also had to rate the empathy they felt for the person and the valence of the emotion they felt. Results showed that amplitude was larger for averted gaze in negative empathy and larger for direct gaze in positive empathy, which indicated that N100 amplitude was modulated by gaze in affective empathy conditions. No effects were found for neutral sentences.

Dozolme et al. [58] explored the P100 component in a study regarding the incongruency effect in sentences and emotional facial expressions. This incongruency effect is believed to reflect the ability to take others’ perspectives and to be associated with higher empathy levels.

Participants were presented with emotional sentences followed by congruent or incongruent FEE of joy, fear, anger, or sadness, as well as neutral faces. They were then asked if the face expressed the same emotion as the sentence. No significant effects were found. In another study [55] involving the distinction between frontal and averted faces and bodies representing happy, angry, and neutral emotions, no correlation was found between P100 amplitude (measured at parieto-occipital sites) and IRI or BEES scores. However, negative correlations were found for P100 latency regarding the IRI-PT for angry averted faces/bodies and happy averted faces, as well as for the IRI-EC and IRI-PD for neutral frontal faces. Finally, in a stop-signal task with happy and fearful faces, a reduced P100 amplitude (at occipital sites) was reported for happy faces in frequent players of video games with graphically violent content at low IRI empathy levels [47]. Empathy scores were also reported to negatively predict P100 amplitude for happy faces in infrequent players. Naumann et al. [38] applied a delayed match-to-sample task, in which children were presented with two successive faces that could be the same (repeated trial) or different (novel trial). Then, the participant had to answer if the faces were the same or not. The authors found no significant correlation between P100 amplitude and the EMK 3–6 empathy scale.

Regarding the N170, the same authors [38] found no significant effect, in line with Dozolme et al. [58], who found no incongruency effect in sentences and FEE content for the N170. While one study [55] points to a negative correlation between N170 amplitude and IRI-EC for angry frontal/averted faces, another found no correlation using the same task and instrument [48]. A negative correlation was likewise found for neutral averted faces and happy, angry, surprised, and afraid faces [57]. Since the N170 is a negative component, these negative correlations represent positive associations, suggesting that greater N170 amplitudes are associated with higher empathy scores.

The N170 latency was also found to be negatively correlated with the IRI-F for averted neutral faces [55]. Regarding amplitude, Choi et al. [57] found a negative correlation with IRI-PT for happy and surprised faces, as well as with IRI-Total for angry and surprised faces, in an oddball task. Contrarily, Bauser et al. [55] found a positive correlation with IRI-PD for frontal angry bodies and averted happy bodies.

Lazar et al. [59] applied an ERP faces–houses task with neutral facial expressions and reported that higher EQ scores predicted a decreased N170 amplitude.

Three studies found no significant correlation with BEES [55] or IRI scores [45,47]. Nevertheless, frequent players of video games with graphically violent content, with lower empathy scores, presented faster N170 latencies for happy than for afraid facial expressions, while infrequent players presented the opposite pattern. McCrackin and Itier [60] found no effect regarding the N170 for eye gaze with empathy elicitation by sentences.

The N170 was studied through an adaptation of the Derntl task [78] intended to explore affective perspective-taking [46], in which the participants were presented with a scenario that could portray an emotional (anger, disgust, fear, sadness, or happiness) or neutral scene. One of the characters in the scenario had their face covered. Then, a target facial expression was presented, which could be congruent or incongruent with the covered expression in the previous scenario, and participants responded as to whether it was an adequate expression or not. In older adults, the N170 amplitude was found to be larger for incongruent than congruent conditions for fear. In a Face Emotion processing task [18], participants were previously presented with pictures of six different actors and introduced to vignettes portraying either intentionally negative, unintentionally negative, or neutral behaviors for each face. Then, pictures of the same actors were presented, expressing different emotions, and the participant had to answer which emotion was shown. Considering the EQ scale, results indicated a significant empathy by character type interaction, corresponding to a tendency for the N170 to present more negative/larger amplitudes for higher empathy scores. This tendency was stronger for intentionally negative and neutral characters than for unintentionally negative characters.

In a study by Luo et al. [42], the P200 amplitude was explored in two tasks with sad and neutral facial expressions, where the participants had to judge the displayed emotion as sad or neutral (an “others-task”) or had to evaluate their feelings in response to the displayed emotions (a “self-task”). A positive correlation was found only in the self-task, in women, between the IRI-PT and the difference wave of sad minus neutral stimuli. In accordance with the findings from studies with the N170, Rodrigo et al. [45] found no correlation between the IRI scores and P200 amplitude. Still, a P200 modulation was reported in both control and neglectful mothers, resulting in an increased positivity for crying expressions in both groups, when compared with laughing and neutral expressions. Altavilla et al. [49] divided a sample into two groups—a control group, who had to spend 7 days writing freely about their day, and an introspection group, who had to write about their emotions and internal motivations for 7 days. The aim was to improve the accuracy of the empathic process, explored through the “Reading the Mind in the Eyes” task [79] performed in the first moment and again following 7 days. This task includes the observation of eye expressions and participants have to select, from four options, which describes the presented mental state. It was expected that the introspection group would present a larger P200 amplitude in the second administration of the task, but no significant effects were found.

Regarding the N200, Balconi et al. [39] divided their sample by BEES scores and found that the high-empathy group presented larger amplitudes for anger, fear, and happiness faces (no distinction was found for sad and neutral faces). However, the directed and averted eye gaze task with previously elicited empathy applied by McCrackin and Itier [60] evoked a more negative N200 amplitude for the neutral condition (e.g., using the verb “fed”) than for positive (e.g., “fed”) and negative empathy (e.g., “killed).

The same study analyzed the EPN and reported larger (more negative) amplitudes for negative empathy than for neutral empathy in the right hemisphere. Finally, Clark et al. [18] also explored the EPN in the face emotion processing task with characters’ stories and found a significant “Character Type by Empathy” interaction. The results indicated that the EPN tends to be larger in participants with very low or very high EQ scores, for all characters—but smallest for those with empathy in the middle range. Furthermore, unintentionally negative characters elicited a larger amplitude in participants with lower empathy scores, while neutral characters elicited larger amplitudes in participants with higher empathy scores.

##### Late Components–N2/P3, P300 N400, LPP, LPC (Late Positive Component), LC1, and LC2 (Late Components)

The N2/P3 Complex amplitude was explored only in one study, with a stop-signal task with happy and fearful faces, which reported no significant empathy effects when IRI scores were included as moderators in post hoc analyses [47].

Regarding the P300 wave, while no significant effect was found between the amplitude elicited by the delayed match-to-sample task with repetitive or novel stimuli and empathy scores [38], the “Reading the Mind in the Eyes” task [79], with eye expressions, elicited a larger P300 to eye expression only in the introspection group, after the participants had to write about their internal states for 7 days (which aimed to improve the accuracy of the empathic process).

In a study conducted by Dozolme et al. [58], who explored the incongruency in emotional sentences followed by congruent or incongruent FEE, as well as neutral faces, the authors included a potential they called the “P3/early N400” (due to time proximity) and the results found were similar to the N400. Specifically, a significant incongruence effect was found in the centro-parietal, parietal, centro-posterior midline, and antero-frontal regions, presenting a larger amplitude for congruent faces, corresponding to more negative components for the incongruent conditions. Higher cognitive empathy scores in the EQ were also found to predict more negative amplitudes for incongruent stimuli in the left occipital region.

The same study found significant effects for the LPP amplitude at the frontal midline and dorso-frontal regions, where incongruent faces elicited a more positive LPP, while in the correspondent electrical counterparts—centro-parietal, parietal, and centro-posterior midline regions—the LPP presented a reduced amplitude to incongruent faces [58].

Rodrigo et al. [45] found no significant correlation between IRI scores and LPP amplitude, but reported that both neglectful and control mothers presented an increased positivity for crying expressions when compared with laughing and neutral expressions, similarly to the P200 (even if the neglectful mothers presented attenuated LPP amplitude across all stimuli compared with the controls). In two studies with oddball tasks, early LPP amplitude was found to be positively correlated with IRI-Total for face targets [56] and IRI-PT scores for angry and afraid faces [57]. The late LPP was reported to be positively correlated with IRI-Total for face targets [56] and happy, angry, surprised, and sad faces [57]. Furthermore, it was also reported to be positively correlated with IRI-F for face targets, non-targets [56], and angry faces [57]; with IRI-PT for happy, surprised, and afraid faces; and with IRI-EC for happy faces [57].

Fernandes et al. [46] found that congruent and incongruent conditions regarding covered expressions from a previous scenario were associated with similar early and late LPP amplitudes in older adults, but not in younger and middle-aged adults, who presented a higher amplitude for congruent than incongruent condition. In the Facial Processing Task with different facial expressions and characterization stories [18], no effect was found for the LPP and EQ scores.

Finally, in the study with the others- and self-tasks with sad and neutral expressions [42], the LPC amplitude was found to be negatively correlated with IRI-PT in the others-task, in women participants, and in the self-task in both men and women. Also, the IRI-F scores were negatively correlated for the others-task in men and for the self-task in women. Altavilla et al. [49] found no introspection effect regarding the LC1 and LC2 amplitude.

#### 3.7.3. Mental States

Four studies explored the perception of others’ mental states, regarding true or false beliefs of an observed person, through scenario observation/description. Information regarding the results for this category can be found in Table 4.

##### Early Components–Early Negative FC (Negative Frontocentral Component)

An early negative frontocentral component was analyzed in a false-belief task [61], in which the participant observed a ball being hidden under one of two shells and was told that the active player (who was, in fact, virtual) could not see the ball after it was hidden. The shells would then rapidly rotate to change positions and there could be a trick, where the ball traded shells without the active player knowing, or no-trick, where the ball never left the original shell. When the shells rotated, this could happen in a velocity considered as “slow” (low difficulty), or three times “faster” (high difficulty). After the rotation, the participant saw the active player choose the shell where s/he believed the ball would be—which could be correct or an error. The results showed that, for low difficulty, higher EQ empathy scores were associated with larger amplitudes for correct trick trials and error no-trick trials.

##### Late Components–Late Negative LC, N400, and LP (Late Positivity)

Albrecht and Bellebaum [61] also explored the late negative LC component in the previously mentioned task, but no significant effect was found. In another study, the same authors [62] applied a similar task, but removed the difficulty condition (slow or faster), meaning that the shell always rotated at the same velocity. Regarding the late negative FC component amplitude, results showed a significant interaction between choice accuracy (correct or error) and trial type (trick or no-trick) only in participants with higher EQ scores. High-empathy participants presented a larger negative amplitude for correct than error in the trick condition, but larger for error than correct in the no-trick condition.

Ferguson et al. [63] applied a task where initial sentences indicated the true or false belief of a character about the location of an object and a second sentence described where the character would look for the object. The second sentence would conclude with a location consistent or inconsistent with the character’s belief. The N400 was explored and it was reported that when the character had a true belief about the location, the N400 amplitude was larger for inconsistent than consistent conditions—the opposite differences were found for false belief. A negative correlation was also found between the N400 amplitude and EQ for the inconsistency effect, but only in false belief.

In another study, the N400 was explored in reaction to four different comic strips—humorous scenes that required ToM; non-ToM humorous strips; non-humorous congruent strips; and non-humorous incongruent strips [64]. The participants only had to observe the strips that were presented and say whether they found them comic or not. A larger N400 amplitude was reported for incongruent, followed by humorous non-ToM, humorous ToM, and congruent strips (smaller amplitude). A positive correlation was found between the N400 amplitude and the Brazilian Empathy Inventory [69] scores in incongruent strips and ToM.

Manfredi et al. [64] found a larger, but negative, mean LP amplitude to incongruent strips, while humorous ToM strips presented the most positive amplitude. Positive correlations with empathy scores were reported in incongruent and ToM strips.

#### 3.7.4. Social Language

Studies in this category explored the perceptions associated with social language aspects, which can be achieved through vocal cues, sentences, and characteristics in lexical sentences. Three studies explored this effect and how it relates to empathy. Information regarding the results for this category can be found in Table 5.

##### Early Components–P100 and P200

Jiang and Pell [65] conducted an experiment to explore the ERP responses involved in a listener’s capacity to evaluate speaker confidence from vocal and verbal cues. Participants heard statements that conveyed three levels of confidence—confident, close-to-confident, and unconfident—followed by lexical congruent phrases. The P100 and P200 components were included in correlation analyses with IRI scores and linear mixed effects models for each ERP with IRI scores as a fixed factor, but no significant effect for empathy scores was reported.

##### Late Components–N400, Late Positivity, Delayed Sustained Positivity, and Late Anterior Negativity

Despite reporting no results on the P100 and N200 components, Jiang and Pell [65] reported an association between IRI total score and N400 amplitude when verbal cues were present, indicating that participants with higher empathy scores displayed a reduced N400 (less negative) to vocal expressions after hearing a lexical phrase.

In a similar study [43], participants heard sentences with a lexical content that could be congruent or incongruent with the probabilistic inferences regarding the speaker’s sex, age, and economic status (e.g., a child’s voice saying that they love to drink wine before going to sleep). Furthermore, besides the speaker identity congruence and incongruence, sentences with lexical semantic congruence and incongruence were presented. Higher empathy scores were correlated with a larger speaker identity N400 effect (the difference between congruent and incongruent conditions), but not for lexical semantics. The EQ was also reported to predict this speaker identity effect.

The N400 was also used to explore how a listener can interpret referential ambiguity in a conversational scenario [66], through the presentation of written interactions conveying speakers and addresses of varying social status and creating Ambiguous, Referent, and Status conditions (per the consistency of the social language used to refer to each person’s social status). Results indicated that participants with higher EQ scores showed larger N400 effects in the Referent condition and that the EQ score predicted the N400 difference between Referent and Ambiguous conditions and between Referent and Status conditions. Jiang and Zhou [66] also reported that EQ scores predicted the effect in the Status condition—subjects with higher EQ scores presented larger Late Positivity and Delayed Sustained Positivity in this condition. EQ scores also predicted the difference between the Status and Referent conditions for the Late Anterior Negativity.

Finally, Jiang and Pell [65] found that participants with higher IRI-Total presented a larger Delayed Sustained Positivity amplitude to utterances with a lack of confidence.

## 4. Discussion

The main objective of this systematic review was to provide an overview of the task designs and ERP components that have been researched in empathy, with a focus on perceptual tasks. Consistent with the previous review conducted by Hall and Schwartz [6], we found a large degree of heterogeneity between studies concerning the concept of empathy, which is reflected in the distinct measures reported and what is considered—or not considered–as empathy in the task designs and conditions.

### 4.1. Design and Methodology

Regarding sample characteristics, it is possible to identify a focus on ages between 18 and 35 years. Several studies did not report the participants’ age range, only one study included children from 4 to 6 years, and none included adolescents. As such, there is a lack of information regarding neurophysiological correlates underlying the development of empathy.

The lack of information about the correlates of empathy in younger populations is crucial, considering that empathic abilities have been identified in childhood, particularly between ages 12 and 16 [1,4]. However, in light of the present review, research in this age group is severely lacking.

As expected, the IRI [7] was the main empathy instrument applied in more than half of the studies that included self-report instruments (in line with the review by Hall and Schwartz [6]). Yet, six instruments were reported and three studies operationalized empathy using only experimental tasks. This could be connected to the referred distinction between what is considered empathy and what is measured, as it has been emphasized that empathy instruments appear to vary in the degree to which they are in accordance with the definition(s) of empathy and among themselves [80].

Focusing on the stimuli, one of the major gaps found in this review is that almost every study used visual stimuli and 14 studies did not report the dataset used to extract the stimuli. This creates difficulties for developing methodology and replication. Replication could help gather information on different instruments using the same procedure and help clarify discrepancies among empathy measures. Furthermore, as previously noted, perceptual processes are not limited to visual stimuli alone [34] and various types of stimuli exert distinct influences on elicited neural correlates [12]. The main emphasis on static visual stimuli restricts a comprehensive understanding of the effects across modalities, channeling research findings predominantly to one direction. For example, audio–visual stimuli, such as videos, are reported to have high emotional state accuracy in EEG tasks [81], potentially constituting a pertinent factor in empathy research. However, none of the reviewed studies incorporated such stimuli.

An important aspect is the identification of four main categories of research, as follows: (1) Affective Pictures; (2) Facial Stimuli; (3) Mental State; and (4) Social Language. All categories are related to different social aspects, allowing for a comprehensive overview of how empathy is intrinsically present in day-to-day life decisions. Despite the possible discrepancies between measurements and concrete definitions, all categories are in accordance with the multidimensional view provided by Decety and Jackson [8] regarding the ability to understand and help others. This should be reflected in similar tasks exploring the same processes, even if some tasks have specific details [12]. However, this is not the case—studies use different task types, conditions, and stimuli to explore the same processes in each of the presented categories (see Section 3 for more details). This could be a useful method if each study was trying to discover something new that had not been explored and further increase knowledge in the field, but most studies had distinct tasks, while trying to explore similar processes.

### 4.2. Event-Related Potentials Findings and Results

The tendency to use inconsistent measures and approaches is also evident in the selection of ERP components, their respective time windows, and sites. Few studies used the same time windows and/or electrode sites, while analyzing the same ERP component. This raises questions about whether the different studies are measuring the same components, attributing the same name to different potentials, or reporting components that are intertwined due to time range overlap [12]. Furthermore, there is variability in the values of filters applied, even when analyzing the same ERP component. Filter cutoffs can result in an alteration in statistical power and distortions of the waveform, contributing to increased heterogeneity [82]. Descriptions of artifact rejection often lack essential details, such as the algorithms utilized for automatic detection, the parameters considered for visual inspection, and reports of included and excluded trials. Adhering to recommended guidelines for filtering settings [82] and signal cleaning [12,83,84] can be a step to lessen these issues.

There is a wide range of EEG/ERP data reported. Eighteen different components were analyzed. As a result, many scattered research findings cannot be statistically compared. Despite all drawbacks and restrictions, the present review attempted to provide the main findings regarding the ERP components and various stages of empathic perceptual processing in each category.

In the Affective Pictures category, early components (N100, P200, and EPN) had no significant correlations with empathy scales, although they were reported to be modulated by positive and negative emotions when compared to neutral, indicating susceptibility to the affective visual aspects. Only the N200 amplitude showed a positive correlation with empathy scores, when humans were presented. The N200 is a negative component that is typically associated with exogenous attention [85], so more empathic subjects would be expected to allocate more attention to others in the early stages of processing, presenting a larger N200 amplitude. However, Groen et al. [41] conducted additional correlations and found that the N200 suppression was positively correlated with larger LPP effects. This could indicate, as the authors stated, “a transition from automatic to controlled processing with a greater N2 suppression taking place in conditions where more attention has to be allocated in preparation of a subsequently more demanding evaluation process (reflected by the LPP)” (p. 153).

In contrast, all late components presented correlations with empathy scales, especially the LPP component, which is thought to reflect sustained attention in the processing of emotionally relevant stimuli [86]. Findings indicate that individuals with higher affective empathy allocate increased and more prolonged attention toward humans expressing positive and negative emotions. This distinction between early and late components could be in line with the referred Decety and Lamm [26] empathy model. All studies but one had either an attention target or questions included in the task, which gives the participant an explicit focus. Despite earlier potentials being sensitive to emotional stimuli, perceptually emotional aspects appear to have been regulated by top-down processes to suppress unnecessary attention in the initial automatic processing stages.

The same pattern is not present in the Facial Stimuli category. Early components present significant correlations with empathy scales and a distinction between negative and positive empathy conditions. For example, it is possible to find correlations suggesting that greater N170 amplitudes are associated with higher empathy, both cognitive and affective. Likewise, late components such as the LPP were positively correlated with measures of cognitive and affective empathy.

However, this is not contradictory with the Affective Pictures results. The Perception-Action Model proposed by Preston and de Waal [27] states that, in early states, automatic processes trigger similar emotional states in the individual when observing others, explaining the modulation of early components. The literature shows that there is an overlap between the brain areas involved in facial and body perception and the brain regions that mediate empathic reactions [15,30]. This means that the visualization of facial and body stimuli automatically initiates similar emotional states and, simultaneously, areas involved in empathy processing, which explains the early modulation of potentials such as the N170 that are connected to facial processing. In the Affective Pictures category, the presented stimuli were more complex, with different scenes, in contrast with individual facial expressions.

The Mental States category focused on the consistency and inconsistency effects associated with empathy scores. All components, especially the N400, presented a larger amplitude associated with empathy scores in inconsistent conditions. This effect indicates that higher empathy levels allow the person to interpret the situation considering the character’s point of view and expect choices following the information the character has—resulting in an effect when this is violated. For example, larger amplitudes for correct responses in trick conditions (without the character knowing), for error in no-trick conditions, and inconsistent behavior in false beliefs. This indicates that more empathic people, despite knowing when a trick or a false belief is in place, update their expectations to others’ views, while less empathic people only consider the information they receive [87]. The study that did not support these results [64] was the only one that did not include others’ perspectives, focusing only on inconsistencies in image sequences.

Yet, the perception of others does not happen only through direct visualization, but also through Social Language. It has vital aspects for communication, such as the ability to establish referential relations [88], provide linguistic content, and transmit relevant information about the speaker through the voice [89]. These are complex aspects that require the processing of social information, depending on later and more top-down processes of predictions and inferences. As such, it is fitting that no significant results were found for early components. The late components reported focused mainly on the N400 component and were in accordance with findings in the Mental State category. It is known that the N400 is sensitive to semantic and linguistic context, especially to mismatched information [90]. However, social language studies show that this effect is associated with empathy traits. Higher empathy scores were associated with larger N400 effects for incongruences in speaker identity. Nonetheless, higher scores were also associated with smaller amplitudes in ambiguous social status interactions (demonstrating sensitivity to underlying contextual information to better adapt to ambiguity) and vocal cues after lexical phrases (suggesting a greater ability to form expectancies about speaker confidence). It appears that, even in the presence of perceptual language aspects, in line with Affective Pictures and faces, empathy allows an optimized integration of social aspects through top-down processes of prediction and representation.

### 4.3. Overview

The overview of the distinct categories provides information on the initial states of empathic perceptual processing, how they vary across distinct tasks, and the different results for each category. In some contexts and stimuli, empathic influences can be identified in early stages (such as Facial Expression), while, in others, they are only present in later stages (such as Mental States and Social Language). Some categories show more varied results, as is observed in the Affective Pictures category.

Design-wise, considering all studies, four main gaps can be identified: (1) the lack of younger samples; (2) inconsistent empathy measures; (3) missing dataset information; and, the most critical, (4) the heterogeneity of the concept of empathy, which is reflected on the different tasks and ERP components to explore the same aspects and processes of interest.

To facilitate the comparison of results across studies, researchers can include the dataset used in each study and uniformize tasks related to the same process. For ERP studies, using the same electrodes and time window to measure the same component would ensure more accurate and comparable results. These modifications are not easy to implement, but a starting point can be achieved through reviews such as the current one. Updated reviews for each category of focus can provide consistent information. Researchers could use these reviews to explore the results associated with their category of focus and obtain information regarding the various aspects of empathy in its perceptual stages—thus allowing a more concise and oriented literature among studies and researchers.

### 4.4. Limitations and Future Considerations

Due to the referred heterogeneity across studies, it was not possible to conduct a meta-analysis. This would provide a more in-depth exploration of the available results. Also, the allocation of studies to broad categories (e.g., Affective Pictures) may be open for debate, as it reflects the interpretations of the authors to provide a clearer review.

In terms of the available literature, limiting inclusion to studies from EBSCOhost, PubMed, and Web of Knowledge databases may restrict access to relevant research in other databases, such as Scopus and IEEE Xplore.

A comprehensive inclusion and codification of the EEG/ERP recording settings and more detailed pre-processing information would be important, to provide a clearer description of common setups. Another limitation is related to the limited number of latency reports. Almost none of the reviewed studies analyzed ERP latencies and, therefore, there is little information on this neurophysiological measure in this review. Similarly, the limited diversity of stimuli modalities, with a predominant focus on static visual stimuli, confines the scope of the review to a primarily visual perception perspective.

Future studies should explore younger samples, to better understand the development of ERPs associated with empathy. These studies should also include latency analysis to explore the temporal aspects of perceptual processes. Additionally, providing detailed information about stimuli, including diversity across modalities, would provide greater replicability of findings and contribute to richer insights. Such enhancements would allow for more organized overviews of the tasks employed and the resulting outcomes.

## 5. Conclusions

This review provides insight into the current state of empathy research with ERP measures regarding perceptual tasks. Distinct categories were identified, allowing an oversight of the main ERP components explored in each one and the differences between them. Results seem to indicate that empathy, even during perceptual states, allows an optimized integration of context information and top-down modulation—helping to better understand perceptual processes that occur across categories.

Several gaps were identified, such as samples, instruments, dataset information, and the heterogeneity of task designs and ERP components. This indicates the need to rethink the present framework to overcome these limitations and provide more unified research on empathy. Researchers in this field would benefit from more detailed information being provided when publishing studies concerning the construct of empathy. Also, while innovative studies looking at different samples, tasks, and ERP components are welcome additions to the scientific literature, this review shows that this approach led to multiple findings that are hard to reconcile. As such, it is also important to ensure that incremental research (using similar tasks and focusing on the same ERP components) is conducted to generate a robust evidence base and ensure the reproducibility of findings. Reviews, such as this one, can be useful during the process of study design, both to identify gaps in the literature and to signal which previous findings warrant confirmation.

## Figures and Tables

**Table 2:** ERP components and results included in the Affective Pictures category.

Latency	ERP Components	Results
**Early Components**	N100 P100 N200 P200 EPN	All ERP components (except the P100) were modulated by emotional stimuli and, in general, presented larger amplitudes for positive and negative stimuli over neutral. Only the N200 component presented a positive correlation with IRI-EC in “Human Effect” and was reported to be smaller for highly negative stimuli than for moderately negative, indicating an affective response to others’ stress.
**Late** **Components**	N300 P300 LPP	All ERP components were sensitive to emotional stimuli. Overall, components presented positive correlations with empathy scales—IRI and BEES. LPP was reported to have positive and negative correlations with IRI-PD.

**Table 3:** ERP components and results included in the Facial Stimuli category.

Latency	ERP Components	Results
**Early Components**	N100 P100 N170 N200 P200 EPN	Heterogeneous results were found amongst components, as follows: **N100**—Modulated by gaze in affective empathy conditions; larger for averted gaze in negative empathy and larger for direct gaze in positive empathy. **P100**—Regarding amplitude, one study found no correlation between IRI and BEES, while another reported that IRI scores negatively predicted the P100 amplitude for happy faces in infrequent players. Negative correlations with IRI-PT, IRI-EC, and IRI-PD for latency. No significant effect was found for repetitive or novel facial expressions and empathy scores in children. **N170**—Positive correlation with IRI-PD; negative correlation with IRI-EC, IRI-PT, and IRI-Total; negative correlation with IRI-F for latency; higher EQ scores predicted decreased N170 amplitude; some studies found no correlation with empathy scales; larger N170 for incongruent than congruent conditions for fear in older adults. **N200**—Larger in subjects with higher empathy scores and for neutral than for positive and negative empathy conditions. **P200**—Positive correlation with IRI-PT. One study found no effect. **EPN**—Larger for subjects with very low or very high EQ scores; larger for unintentionally negative characters in lower EQ scores and for neutral characters in higher EQ scores; larger for negative than neutral empathy condition (right hemisphere).
**Late** **Components**	N2/P3 P300 N400 LPP LPC LC1 LC2	No significant effect was found for the P300 regarding repetitive or novel facial expressions and empathy scores in children. Larger P300 to eye expressions in the introspection group after a 7-day writing task, but not in the control group. Higher EQ scores predicted larger N400 amplitudes. **LPP**—Correlations with IRI-Total, IRI-PT, and IRI-F; larger for congruent than incongruent covered faces in younger and middle-aged adults. No significant interaction between LPP and EQ. LPC was reported to be positively correlated with IRI-PT and negatively correlated with IRI-F. No significant effects were found for LC1 and LC2.

**Table 4:** ERP components and results included in the Mental States category.

Latency	ERP Components	Results
**Early Components**	Early Negative FC	For the early negative FC component, amplitude in subjects with higher EQ was modulated by choice accuracy—larger for correct in the trick condition and for error in the no-trick condition.
**Late** **Components**	Late Negative FC N400 LP	For the late negative FC component, amplitude in subjects with higher EQ was modulated by choice accuracy—larger for correct in the trick condition and for error in the no-trick condition. Another study found no effect. N400 amplitude was negatively correlated with EQ scores for inconsistency effect—but only in false belief. N400 and LP amplitudes were positively correlated with empathy scores for incongruent strip condition. However, N400 amplitude was larger for incongruent, humorous non-ToM, and humorous ToM conditions, while LP amplitude was larger for humorous ToM.

**Table 5:** ERP components and results included in the Social Language category.

Latency	ERP Components	Results
**Early Components**	P100 P200	No effects were found for the P100 and P200 components.
**Late** **Components**	N400 Late Positivity Delayed Sustained Positivity Late Anterior Negativity	**N400:** Reduced amplitude for vocal expressions after lexical phrase in subjects with higher empathy scores; higher EQ scores were associated with larger N400 for speaker identity effect (difference between congruent and incongruent conditions) and Referent condition. **Delayed Sustained Positivity:** Larger amplitude to utterances with lack of confidence and for Status condition in subjects with higher empathy scores. Higher empathy scores were associated with larger Late Positivity in Status conditions and with Late Anterior Negativity amplitude difference between Status and Referent conditions.

## Data Availability

No new data were created or analyzed in this study. Data sharing is not applicable to this article.
